# Scientific perspectives on lunar exploration in Europe

**DOI:** 10.1038/s41526-023-00298-9

**Published:** 2023-06-24

**Authors:** Jessica Flahaut, Carolyn H. van der Bogert, Ian A. Crawford, Sebastien Vincent-Bonnieu

**Affiliations:** 1 CRPG, CNRS-UMR7358/Université de Lorraine, 54500 Vandœuvre-lès-Nancy, France; 2 Institut für Planetologie, Westfälische Wilhelms Universität-Münster, Münster, Germany; 3 Department of Earth and Planetary Sciences, Birkbeck College London, Malet Street, London, WC1E 7HX UK; 4 ESA-ESTEC, Noordwijk, Netherlands

**Keywords:** Physics, Scientific community

## Abstract

The Moon is a geological history book, preserving information about the history of the Solar System, including the formation and early evolution of the terrestrial planets and their bombardment histories, as well as providing insight into other fundamental Solar System processes. These topics form the basis for science “of the Moon”, but the lunar surface is also a platform for science “on the Moon” and “from the Moon”—including astronomical observations, fundamental physics, and life science investigations. Recently, the Moon has become a destination for technology research and development—in particular for developing in situ resources, human exploration, and habitation, and for its potential use as a waypoint for the human exploration of Mars. This paper, based on recommendations originally proposed in a White Paper for ESA’s SciSpacE strategy, outlines key lunar science questions that may be addressed by future space exploration missions and makes recommendations for the next decades.

## Introduction

The Moon is a high-priority target for exploration by the world’s space agencies^1^. In this context, key lunar science questions have been compiled, discussed, and reviewed by the lunar science community in the course of several major studies. In particular, the American National Research Council (NRC) published a report entitled “Scientific Context for Exploration of the Moon”^2^, which has served as a reference for experiment, mission, and strategic planning since its publication in 2007. Recently, the Lunar Exploration and Analysis Group (LEAG) reviewed the progress made in achieving the goals outlined in the NRC report^3^, where they retired some goals and added new goals based on the results and questions arising from recent studies and missions. These and other reports and papers (e.g., refs. ^4,5^) were used to support the definition of ESA’s scientific and technological strategies for the Moon^6,7^. The present paper, based on recommendations originally proposed in a White Paper for the European Space Agency’s (ESA) SciSpacE strategy^8^, represents the most up-to-date effort from the European planetary science community to summarize the existing documentation and formulate recommendations. Outstanding lunar science questions are summarized below and in Table 1.

**Table 1:** Open scientific questions (in bold) and proposed recommendations from ref. ^8^.

Open fundamental scientific question	Future space experiments	Relevance for space exploration	Recommendations (short (S), medium (M), and long (L) term)
**Bombardment in the inner Solar System** Ancient bombardment: Nature of bombardment on the early Moon? Was there a lunar cataclysm or planet migration in the early Solar System? Relation to the emergence of life on Earth? More recent bombardment: Understand the nature of bombardment in the last 1 Ga—constant or in swarms? Present bombardment: What is the present flux? - occurrence of meter category objects in the near-Earth region	In situ and/or sample return age analyses for (1) key lunar basins (including South Pole-Aitken), (2) young basalts, (3) key young craters Remote observations of newly formed craters, and installation of seismic networks to measure their frequency and magnitude	Present bombardment: assess present hazard for the Earth and also specifically for lunar surface operations Absolute chronology for the Solar System: Improving the absolute chronology is critical for interpreting the geological history of all terrestrial planetary bodies to which it is applied; this fundamental information feeds into agency strategy and mission planning Technology driver: Development of communications, landing, payload, and surface operations capability	(S) Participation in Chang’e-5 and Chang’e-6 sample analyses (S) Develop/support implementation of seismic/geophysical payloads (M) In situ analysis payload development, sample return mission or payload development for EL3 (M-L) At least one in situ age or sample return mission to a key geological unit (L) Gateway-based, human/robotic sample return concepts
**Lunar interior, seismicity, tectonics** Steps of formation/ differentiation of the Moon? Origin of subsequent asymmetric thermal and volcanic evolution? Current level of seismicity?	Globally distributed seismic and heat-flow network; expanded retroreflector network	Evaluate present hazard for surface operations/construction Technology driver: Development of communications, landing, payload, and surface operations capability	(S) Develop/support payloads with geophysical instrumentation (S) Support placement of geophysical network nodes at the mission of opportunity sites (L) Deploy geophysical network across the Moon
**Geological processes as revealed by the Moon** Formation/evolution of crust: Crustal homogeneity (LMO, KREEP etc.)? Volcanism: How recent? Role of volatiles? Thermal evolution? Interior diversity? Resources? Impact cratering: Processes as revealed on a body with less active geology than the Earth?	Sample return and in situ measurements, chronology, and mineralogical/geochemical compositional measurements Orbital compositional observations at higher resolutions and expanded wavelength ranges Regional seismic networks for understanding subsurface structures (e.g., lava caves) Observation of changes over time (tectonics/impacts/ mass-wasting)	Resource assessment for materials applicable to ISRU, construction, and other surface activities Scientific input for agency strategy and mission planning Technology driver: Development of communications, landing, payload, and surface operations capability	(S) Participation in Chang’e-5 and Chang’e-6 sample analyses (S) Develop/support placement of basic mineralogical/geochemical packages on missions of opportunity (S) Support additional studies of landing sites for assessment of science questions, including hazard assessment and specific site selection, for agency-directed use during missions of opportunity (M) Remote observation of lunar surface at extended thermal infrared wavelengths (M) Support/develop follow-on mission for NASA’s Lunar Reconnaissance Orbiter mission/ShadowCam—like instrument to provide high resolution imaging of the lunar surface (M) In situ analysis payload development, sample return mission or payload development for EL3 (L) Gateway-based, human/robotic sample return and analysis concepts
**Water and other volatiles** Origins? Distribution? Abundances? Compositions? Processes? Lunar volatile cycles? Resources?	Orbital observations and ground truth of nature, distribution, extent, composition of water and volatiles Cores inside the regolith to investigate vertical distribution and nature Cryogenic sample return	Resource assessment for materials applicable to ISRU, construction, and other surface activities Technology driver: Development of communications, landing, payload, and surface operations capability; Development of ice/volatile handling and storage	(S/M) PROSPECT on CLPS and other CLPS opportunities, Viper, similar missions (M) Support delivery of analytical/technical payloads to polar regions (M) Orbital mapping of H_2_O ice and other species at an unprecedented resolution (for instance, with LunaH-Map like cubesats) (M) Collaborate with CNSA on lunar research station at lunar south pole (M) ESA EL3 (L) Cryogenic sample return; multiple drill cores (L) Gateway-based, human/robotic sample return and analysis concepts
**Regolith** Formation and weathering processes? History of the Sun and Solar System? Resources?	Sample return, regolith stratigraphy /deep drill core, samples of paleoregolith; swirls	Resource assessment for materials applicable to ISRU, construction, and other surface activities Technology driver: Development of communications, landing, payload, and surface operations capability; Development of deep-drilling technologies	(S) Participation in Chang’e-5 and Chang’e-6 sample analyses (S) Participation in analyses of recently opened Apollo samples (S) Develop/support placement of basic mineralogical/geochemical/geotechnical packages on missions of opportunity (M) ESA EL3 (M/L) Sample return, return of cores and paleoregolith (L) Gateway-based, human/robotic sample return and analysis concepts
**Atmosphere, dust, and plasma environment** Exosphere formation and evolution? Dust levitation and transport? Sources of mid-latitude surface hydroxyl and water? Migration of hydrogen to cold traps? Electrostatic lofting of dust associated with plasma anomalies/voids? Changes due to surface activities?	UV and mass spectrometers, material adhesion experiments, Langmuir probes	Assess hazards for future robotic and human exploration	(S) Develop/support placement of plasma, neutrals, magnetic and electric fields and dust particles instruments and follow-on LADEE-like experiments on missions of opportunity (M-L) Deploy a global network of long-term monitoring stations (including ion-mass spectrometers, optical UV spectrometers, dust and plasma experiments)
**Moon as a platform** Astronomy, astrophysics, fundamental physics, life sciences and astrobiology, e.g., adaptation of life (including human physiology) to low (but non-zero) gravity and enhanced radiation environments	Low-frequency radio-antennas, optical/IR telescopes, laser retroreflectors, cosmic ray detectors combined with boreholes; bio-regenerative life-support systems; agricultural systems; radiation protection; mitigation of long-term exposure to low gravity	Prepare for long-term human habitation of the Moon and human missions to Mars and beyond	(S) Deploy laser reflectors on near-term robotic landers (M-L) Deploy farside low-frequency radio antennae; deploy cosmic ray detectors; deploy Earth-observing instruments (including instruments to study Earth’s magnetosphere); deploy life sciences experiments (L) Gateway-based, human/robotic concepts

Recommendations are proposed in the short (10 years) term.

## Bombardment history of the inner solar system

The lunar surface provides a largely complete record of the impact history of the Earth-Moon system throughout Solar System history^9^. Several aspects of this record are of compelling scientific interest, including whether or not there was a spike in the impact rate about 3.8 billion years ago (the so-called Late Heavy Bombardment)^10^, and whether the impact flux since then has been mostly constant or has included episodic spikes^11^. An understanding of the modern impact history of the Moon is also important for informing estimates of the current terrestrial impact hazard. To this end, the lunar surface is an ideal target for estimating the current impact rate, as ongoing impacts can be monitored by impact flashes, and further coupled to the statistical analysis of the smallest craters and recent events (e.g., refs. ^12,13^). The lunar impact record has been partially calibrated by measuring crater size-frequency distributions (CSFDs) or spatial densities on surfaces for which laboratory radiometric or exposure ages have been derived from returned samples. These chronology functions are used, with various assumptions, to estimate the ages of cratered surfaces throughout the Solar System. However, the calibration of the chronology function mostly relies only on samples with ages of <1 billion and between 3.2 and 3.9 billion years old. The only samples of well-established provenance with ages between ~1 and 3 Ga are the ~2.0 Gyr-old samples recently returned by the Chang’e-5 mission^14^. As noted in previous reports (e.g., ref. ^4^), improving the calibration of the lunar cratering chronology is of great value for planetary science because it would: (1) provide better estimates for the ages of unsampled regions of the lunar surface; (2) give a more reliable estimate of the impact history of the inner Solar System, especially that of our own planet; and (3) enable better estimates for the ages of other planetary surfaces (e.g., that of Mars) from which samples have not yet been obtained. In order to meet these objectives, it will be necessary to visit geological units—e.g., ancient impact basins and young basalts—of a much wider range of ages than previously sampled and either return samples to Earth for radiometric dating or precisely radiometrically date the materials in situ. Activities that have been rated as highest priority in previous strategic documents include: (1) investigate the occurrence of an early impact spike or cataclysm, (2) determine the age of the oldest lunar basin (South Pole-Aitken basin), (3) better calibration the lunar cratering chronology, and (4) constrain the current impact flux.

## Interior structure of the moon and lunar seismicity

Improved understanding of the interior structure of the Moon will provide fundamental information on the evolution of differentiated planetary bodies. The Moon is especially important in this respect because the absence of internal activity associated with mantle convection (such as plate tectonics on the Earth) means that the interior of the Moon likely retains a record of early planetary differentiation processes (e.g., magma ocean crystallization) that are no longer preserved in more evolved planetary bodies. Understanding the history of lunar magnetism, and its relationship to the evolution of the lunar core, will also provide insights into the mechanisms of magnetic field generation in terrestrial planets (e.g., ref. ^15^). Previous reports^2,3,6^ outline the following scientific goals: (1) investigation of the thickness and lateral variability of the lunar crust (and of the nearside/farside crust asymmetry), (2) investigation of the chemical and physical stratification of the mantle, (3) characterization of the lunar core, and (4) determination of the current thermal state of the lunar interior. Making progress in these areas will require deploying geophysical instruments to the lunar surface. Key instruments include seismometers to probe the structure of the deep interior, heat-flow probes to measure the heat loss from the lunar interior and its spatial variations, magnetometers to measure the thermal conductivity profile and local surface magnetic fields, and laser reflectors to measure the Moon’s physical librations which are related to the distribution of mass in the interior. Such measurements may also yield data relevant to studies of gravity and fundamental physics.

**Fig. 1:**
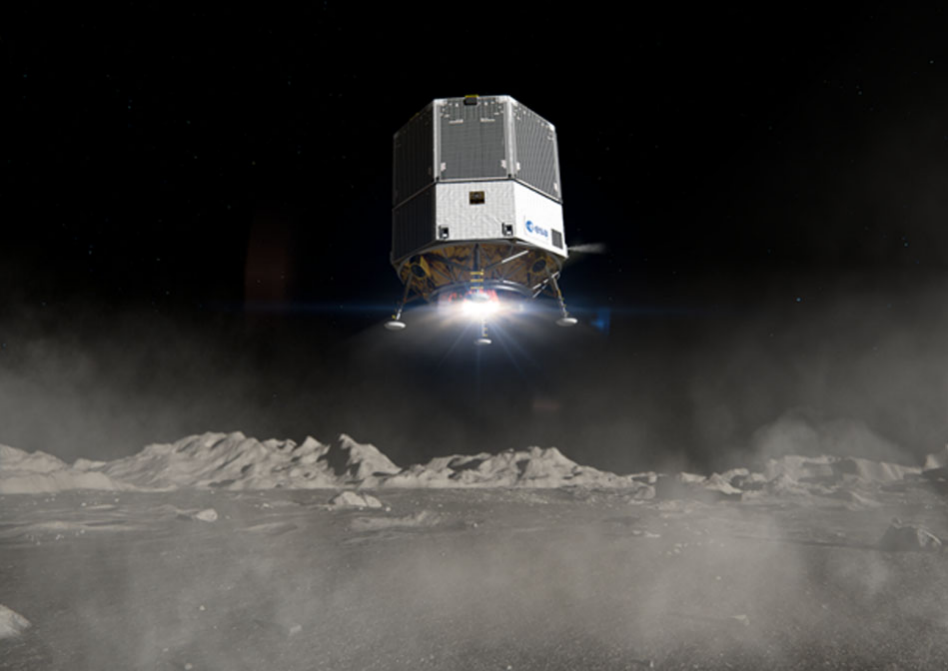
Artistic view of the European Large Logistic Lander, Argonaut, delivering supplies to the lunar surface (courtesy of ESA).

Ideally, a lunar geophysical network could be assembled by equipping multiple landers (e.g., ESA’s proposed ‘Argonaut’ European Large Logistic Lander (EL3), (Fig. 1) and/or commercial missions of opportunity) with a standard set of geophysical instruments including a seismometer, heat-flow probe, magnetometer, and laser reflector^16^. Studies of lunar magnetism would additionally benefit from the collection of samples from which remanent magnetic fields, including evidence for paleofield orientations and reversals, could be measured^17^. Such sample collection is likely to benefit from a human presence on the lunar surface, which would also facilitate the deployment of a wider range of geophysical instrumentation than could be deployed robotically.

Continued high spatial resolution imaging of the lunar surface would allow further identification of small tectonic features on the Moon and allow the assessment of the current seismic activity and hazards. Due to the fresh morphology of some small lunar scarps (<100 m in relief) and related graben, it is possible that moonquakes might be associated with their formation^18^. We note however that ongoing tectonic activity near active scarps and wrinkle ridges (e.g., ref. ^19^) may pose a hazard for robotic and human surface activities.

## Lunar geological processes

In addition to processes specifically addressed above, other high-priority geological goals include investigations of: (1) the formation and evolution of the crust, (2) lunar volcanism, and (3) the impact cratering process.

**Fig. 2:**
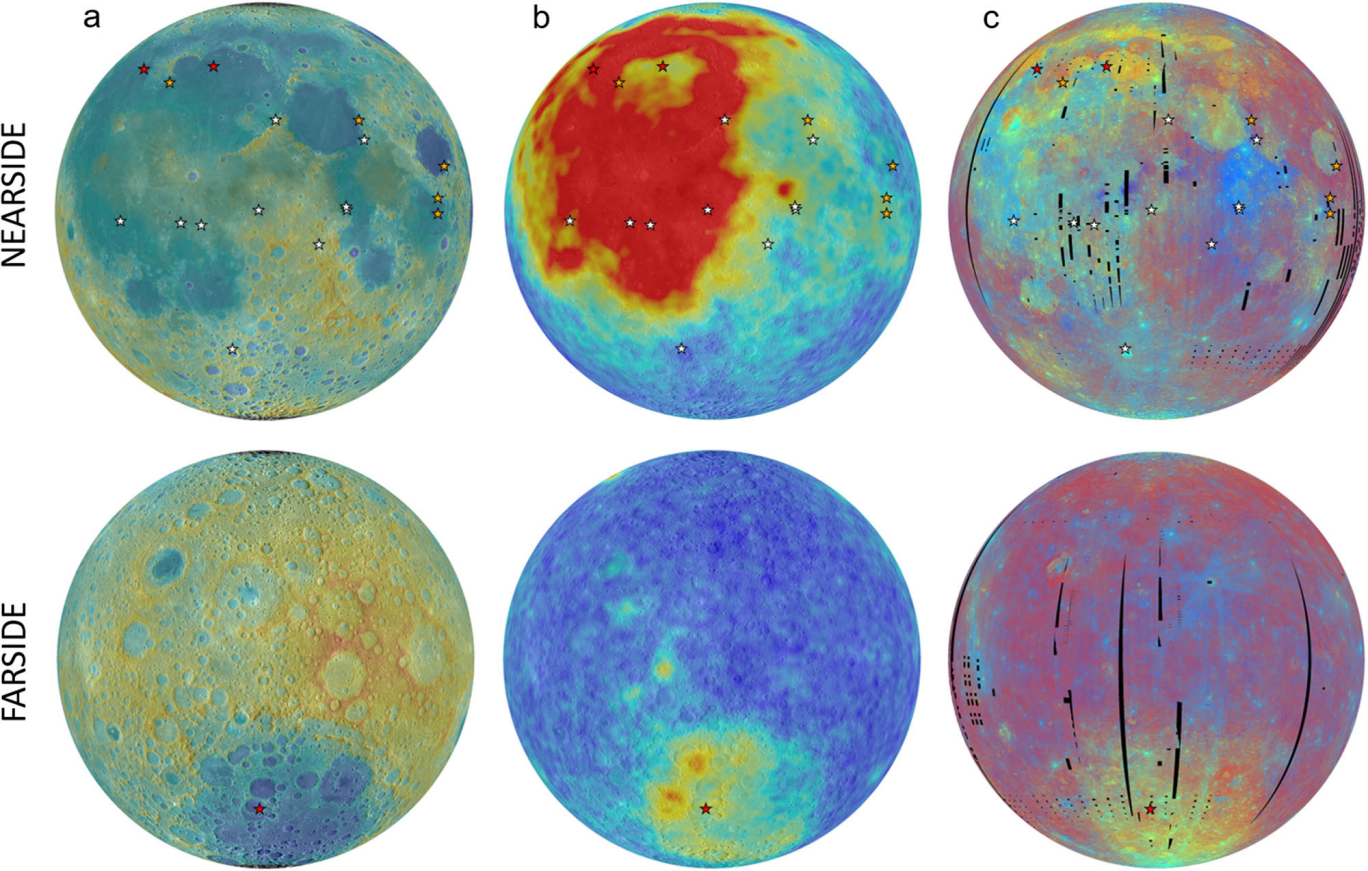
Modern views of the lunar surface. **a** Topography from the LRO WAC GLD100 Digital Terrain Model (rainbow scale, −8.5 to +10.5 km). **b** Thorium abundance from the Lunar Prospector gamma-ray spectrometer (rainbow scale, 0–13 ppm). **c** Clementine UVVIS color ratio mosaic. Colored stars indicate the location of previous successful surface missions; White = United States of America (Apollo and Surveyor; note the symbols representing Surveyors 3 and 5 overlap with those of Apollos 12 and 11, respectively), Orange = Soviet Union (Luna), Red = China (Chang’e).

The rich remote sensing data collected over the last decade has improved the understanding of lunar crustal rock compositions and mineralogy, as well as their distribution across the Moon (e.g., Fig. 2^4,20–22^). With these data as a basis for selection of targets, for higher resolution and/or additional wavelength-range remote observations, and landing sites, for in situ analyses and sample collection/return, we can gain more information about key planetary processes that are revealed in the lunar crustal rocks (e.g., refs. ^2,23^). Goals outlined in earlier reports include: (1) investigating the compositions and distributions of the feldspathic crust, KREEP terrane, lower crustal materials, and the bulk Moon; (2) identification of new lunar rock types and their ages, distributions, and origins; (3) determining the local and regional complexity of the crust; and (4) exploring the vertical extent and structure of the megaregolith. Investigations of lunar crustal rocks can be driven forward by more advanced remote sensing measurements, in situ and returned sample analyses, and also by the installation of seismic stations and networks.

Volcanism is a major geological process on the Moon, which has significantly shaped the current lunar surface, and also provides information about its compositional and thermal evolution^24–26^. Important questions regarding lunar volcanism include the determination of: (1) the origin and variability of lunar basalts; (2) the ages of the youngest and oldest basalts (which is also relevant for calibrating the lunar cratering chronology); (3) the compositional range and extent of pyroclastic deposits; and (4) the style and diversity of lunar volcanism (including Irregular Mare Patches and silicic domes generation (e.g., refs. ^27,28^), (5) the volcanic flux and evolution^2,6^. Studies of lunar volcanism not only feed into the lunar cratering chronology, but also aid in the understanding of the nature and evolution of the lunar interior. Advanced remote sensing measurements, in situ analyses, and sample return missions can all contribute to the achievement of volcanism-related science goals.

Due to the absence of a substantial atmosphere, liquid water, and plate tectonics, the Moon also provides important information about impact cratering processes—from micro-impacts to basin-sized impacts (e.g., refs. ^9,29^). Specific major goals for understanding impact cratering processes on the Moon include investigating: (1) impact melt sheet differentiation; (2) multi-ring impact basin structure; (3) the influence of planetary properties on crater formation and morphology; and (4) the extent of mixing of materials both proximal and distal to craters. Again, remote sensing, in situ analyses, and sample return missions, as well as geophysical observations, will provide input for these issues.

## Regolith processes

The lunar regolith is a several meters-thick layer of unconsolidated material which covers the lunar surface, and includes a mixture of both lunar soils (<1 mm) and rock fragments. The regolith, which is mainly formed by meteoroid bombardment of the surface, also collects solar wind particles and the cosmogenic products of galactic cosmic rays, and therefore retains a unique record of solar and galactic events over billions of years^30,31^. The regolith will also be the prime source for derivation of in situ oxygen, water, metals, and other important volatiles and building materials^32,33^. After previous landed and orbital missions, a number of questions pertaining to (1) the formation mechanisms of regolith on airless bodies, (2) its modification (space weathering, deposition of volatile materials), (3) its physical properties (strength, cohesion, composition, grain size), and (4) its lateral and vertical variations remains. Analyzing and collecting regolith from various locations outside of the to-date investigated areas at the Apollo, Luna, and Chang’e landing sites—in particular, polar locations, feldspathic highlands, young terrains, pyroclastic deposits, and lunar swirls—would enhance our understanding of regolith properties and variability. Missions with mobility elements increase the opportunities to (1) understand and characterize lateral variations, (2) search for and investigate ancient regolith (and hence the ancient Solar System record^34^), and (3) identify and study rare materials in the lunar regolith, possibly including meteorites derived from the early Earth^35^.

## Lunar volatiles

Both sample analyses and orbital remote sensing observations point to the existence of water in various forms at the lunar surface (e.g., refs. ^36–39^). Indigenous water has been found in lunar minerals^36^, whereas solar wind is thought to be responsible for diurnally variable hydroxylation and hydration of the lunar surface by exogenous processes^37^. With annual temperatures as low as 40 K, some high-latitude areas which never receive sunlight (Permanently Shadowed Regions or PSRs) are expected to concentrate and retain H_2_O ice along with numerous other volatiles (e.g., CO_2_, NH_3_, SO_2_^38^). Lunar polar volatiles trapped in PSRs near the poles may hold clues to the origin of water in the inner Solar System and also have strong potential as a reservoir for extraction of water, oxygen, and other volatiles^7,32,33^. The origin, vertical and lateral distribution, abundance, resource potential, age, and transportation/accumulation cycle of lunar volatiles are all poorly constrained, and could be addressed with both in situ measurements and/or sample return from the polar regions. Non-polar volatiles could also be analyzed through sample return missions targeting specifically (1) equatorial mid-latitudes regions inside and outside of lunar swirls, to assess the role of solar wind implantation, (2) freshly exposed/young material which has not been affected by long-term solar wind exposure, and (3) volcanic provinces and pyroclastic deposits which may exhibit enhanced indigenous volatiles contents.

## Atmosphere, dust, and plasma environment

The lunar atmosphere is the most accessible surface boundary exosphere in the Solar System, and thus offers insights into surface sputtering, meteoritic vaporization processes, exospheric transport processes, and gas-surface thermal and chemical equilibration [ref. ^40^ and references therein]. This dynamic system also plays a role in the transportation and deposition of volatile elements^41^. Still, its composition, sources (comets, asteroidal meteorites, transit of interstellar giant molecular clouds, Earth, Moon interior), sinks (photodissociation, Jeans escape, solar wind pickup, condensation), and variations due to impacts, diurnal cycles, and solar activity are poorly understood. Models show that once released by heating or sputtering, atmospheric volatiles migrate towards the poles where they are trapped in PSRs (e.g., refs. ^41,42^), but further measurements are needed to determine what processes control the atmospheric migration and the efficiency of the transport to the poles. The fragile lunar atmosphere should be characterized with instruments such as ion-mass spectrometers and optical/UV spectrometers onboard low-altitude orbiters or landers in the short-term before surface activities further perturb it from its native state. In addition to He, Ne, and Ar, lunar dust is a major component of the lunar atmosphere. Moon dust is formed by micron and sub-micron-sized particles charged by the local plasma environment and/or ejected by micrometeoroids, and can travel via two mechanisms: levitation and lofting^43^. Conjectured transport phenomena range from the levitation of micron-size dust grains at low altitudes (centimeter to meter height) to the lofting of sub-micron particles to tens of kilometers. As lunar dust is surprisingly abrasive, understanding its physical properties (size, charge, distribution) is key to future exploration. Dust impacts were mostly observed to peak around the terminator region, suggesting a relationship with horizon glow^43^, and illustrating the necessity to better understand the electric potential at the lunar surface and the dust/plasma interactions with the deployment of experimental packages at a network of monitoring stations.

## The moon as a platform for scientific investigations

In addition to its intrinsic interest to planetary science, the Moon is also a potential platform from which a diverse range of scientific investigations (e.g., in astronomy and astrophysics, in life sciences) may be supported (e.g., refs. ^6^^,44–46^).

One of the principal benefits of a lunar platform for astronomy is the usefulness of the radio-shielded farside for low-frequency radio astronomy^44,45^. Radio waves with wavelengths longer than about 20 m cannot penetrate the Earth’s ionosphere, and so must be observed in space. These wavelengths are expected to be a rich source of astrophysical information—including highly red-shifted 21 cm lines absorbed against the cosmic microwave background by hydrogen clouds shortly after the Big Bang^44^. The lunar farside is probably the best location in the Solar System from which such observations could be made. Observations at all other wavelengths could also be made from the lunar surface. Although to-date many such observations are made from free-flying spacecraft, the lunar surface may still offer some advantages (e.g., the possibility for passive cooling of IR instruments in permanently shadowed lunar craters, and the provision of a solid substrate on which to mount optical/IR interferometers (e.g., refs. ^45,47,48^). Moreover, in the context of ESA’s Exploration Programme^6^, access to the infrastructure provided by human activities on the lunar surface would aid in the maintenance and upgrading of astronomical instruments compared to free-flying satellites. Finally, the lunar surface lends itself to studies on the interface between astrophysics and fundamental physics (e.g., by facilitating emplacement on the lunar surface of instruments to study ultra-high-energy cosmic rays, general relativity, and quantum entanglement over the Earth-Moon baseline).

The Moon is also a potential laboratory for understanding the environmental parameters that affect life in space^46^. For example, the Moon can be used to investigate the biological effects of: low, but non-zero gravity, the radiation environment beyond the Earth’s magnetosphere, and the toxicity of lunar dust. A diverse range of organisms could also be taken to the lunar surface and used to carry out investigations in situ. These experiments would yield new insights into fundamental biological processes and the adaptation to, and evolution of, organisms in the space environment. This would feed into the implementation of bio-regenerative life-support systems, food production, and the mitigation of adverse consequences of low gravity and high radiation environments. These studies may help enable human exploration elsewhere in the Solar System, for example, the surface of Mars.

## Priorities for the space programme

Key steps for future long-term lunar exploration will require the development of enabling technologies, including:

- Precision landing and automated hazard avoidance
- Surface mobility
- Power and heating systems to enable survival during lunar night and within PSRs
- Satellite network to ensure continuous communication with missions on the farside or radio-shadowed areas (e.g., PSRs)
- Tele-robotics
- Significant (>1000 kg) landed payload masses
- Deep (10–100 m) drilling capability
- Sample return
- Cryogenic sampling and caching
- Human operational capabilities in the lunar vicinity and/or on the lunar surface.

The list in this section is not exclusive but serves to highlight areas for technology development that are foreseen to achieve key scientific investigations in the next decades. It builds on foreseen achievements in the next years which should result from international missions such as NASA’s CLPS and Artemis programs, on which ESA is collaborating. As outlined in Table 1, short-term progress can be made in answering fundamental scientific questions about the Moon by leveraging existing technological capabilities and partnerships. Plans for mid-to-long-term strategic technology development, including the capabilities listed above, will enable the achievement of higher-level scientific goals. Many of the scientific goals can be accomplished via remote sensing and robotic missions. However, detailed geological studies and refined selection of lunar samples will benefit significantly from the involvement of astronauts, either via tele-robotics in the lunar vicinity or on the lunar surface^49^. A station or Gateway in the lunar vicinity could serve as a platform for lunar surface operations, including remote collection and transfer of lunar samples^50^. A human presence on the Moon will not only allow the dexterous and dynamic collection of samples and measurements for investigation of scientific questions in real-time, but also provide a valuable testbed for technologies that enable exploration of more distant destinations.

Development of key payloads and technology drivers is expected to enable robotic and human exploration beyond low Earth orbit, in the vicinity of the Moon and on the lunar surface. Technologies developed during lunar-related activities will provide valuable input for missions to worlds more distant. For example, precision landing and automated hazard avoidance, tele-robotics, and surface mobility improvements can be applied to missions to all solid planetary bodies. Development of cryogenic sample return could be used on missions to comets and icy moons in the outer Solar System. ISRU and lunar construction technologies will serve as a basis for longer-term habitation of the Moon and foster a lunar economy to support further research and development. Moreover, technological developments in robotics, communications, resource use, and other technologies will feed back to terrestrial applications.

## Outlook and summary

Over the last half century, robotic lunar missions have driven significant advances in our understanding of the nature, formation, and evolution of our Moon, as well as the Earth and other planetary bodies. These advances have led to new, more refined questions that require more technologically-advanced observations and measurements, and the collection of additional samples that are not yet represented in our current collection. The accessible and unique ~4.5 billion year geological record preserved on the Moon is a treasure that will reveal fundamental discoveries, not just about the Earth-Moon system (including the habitability of our own planet), but also about the geological processes involved in planetary formation and evolution more generally. In addition, there are growing international opportunities to use the Moon as a platform for other scientific investigations (e.g., in astronomy, biology, medicine, and physics), for development of in situ resources, and as a gateway to more distant worlds such as Mars. We have not attempted to rank specific scientific questions in order of priority; rather, each topical area is accompanied by short, mid-, and long-term technological goals and recommendations. Thus, a combination of scientific questions can be addressed within the strategic framework of current and future space exploration technologies. Nevertheless, progress in development of new and more capable technologies is a requirement for achieving higher-level scientific goals. The synergies between scientific and technological exploration strategies for the Moon promise exciting advances in the next decades, including the return of humans to the lunar surface.

## Data Availability

No data was generated throughout the manuscript. Global datasets used to produce Fig. 2a, c are publicly available on the USGS Astropedia website: https://astrogeology.usgs.gov/search?pmi-target=moon. Lunar Prospector gamma-ray spectrometer data from Fig. 2b are available from the NASA Planetary Data System: https://pds-geosciences.wustl.edu/missions/lunarp/reduced_grsns.html.
